# Tlr1612 is the major repressor of cell aggregation in the light-color-dependent c-di-GMP signaling network of *Thermosynechococcus vulcanus*

**DOI:** 10.1038/s41598-018-23628-4

**Published:** 2018-03-28

**Authors:** Gen Enomoto, Yukiko Okuda, Masahiko Ikeuchi

**Affiliations:** 1 Department of Life Sciences (Biology), Graduate School of Arts and Sciences, The University of Tokyo, Komaba 3-8-1, Meguro, Tokyo 153-8902 Japan; 2 Core Research for Evolutional Science and Technology, Japan Science and Technology Agency, 4-1-8 Honcho Kawaguchi, Saitama, 332-0012 Japan

## Abstract

Cyclic diguanylate (c-di-GMP) is a bacterial second messenger involved in sessile/motile lifestyle transitions. We previously reported that c-di-GMP is a crucial inducer of cell aggregation of the cyanobacterium *Thermosynechococcus vulcanus*. The three cooperating cyanobacteriochrome photoreceptors (SesA/B/C) regulate cell aggregation in a light color–dependent manner by synthesizing/degrading c-di-GMP. Although a variety of c-di-GMP signaling proteins are encoded in cyanobacterial genomes, how c-di-GMP signaling networks are organized remains elusive. Here we experimentally demonstrate that the cellulose synthase Tll0007, which is essential for cell aggregation, binds c-di-GMP although the affinity is low (K_d_ = 63.9 ± 5.1 µM). We also show that SesA—the main trigger of cell aggregation—is subject to strict product feedback inhibition (IC50 = 1.07 ± 0.13 µM). These results suggest that SesA-produced c-di-GMP may not directly bind to Tll0007. We therefore systematically analyzed all 10 of the genes encoding proteins containing a c-di-GMP synthesis/degradation domain. We identified Tlr1612, harboring both domains, as the major repressor of cell aggregation under the repressing teal-green light irradiation. *tlr1612* acts downstream of *sesA* and is not regulated transcriptionally by light color, suggesting that Tlr1612 may be involved in c-di-GMP amplification in the signaling cascade. Post-transcriptional control is likely crucial for the light-regulated c-di-GMP signaling.

## Introduction

Cyclic diguanylate, or bis-(3′-5′)-cyclic diguanylic acid (c-di-GMP) is a second messenger that is most commonly found in bacteria. C-di-GMP was originally identified as an allosteric activator of cellulose synthesis in *Komagataeibacter xylinus*^1,2^. Subsequent studies revealed that c-di-GMP generally induces sessile, multicellular lifestyles such as biofilms, and that it represses the motile, planktonic lifestyle in various bacteria^3,4^. C-di-GMP is also involved in cell-cycle progression and the expression of virulence genes, indicating that c-di-GMP orchestrates various cellular responses to effect drastic changes in bacterial physiology. C-di-GMP is synthesized by the diguanylate cyclase (DGC) activity of GGDEF domains^5^ and degraded by the c-di-GMP–specific phosphodiesterase (PDE) activity of EAL and HD-GYP domains^6,7^. C-di-GMP binds to and regulates various effectors such as proteins containing a PilZ domain^2,8,9^ or a MshEN domain^10^, transcription factors^11^, and riboswitches^12,13^. Notably, bacterial genomes often contain multiple genes encoding DGCs and c-di-GMP PDEs that encode GGDEF/EAL/HD-GYP domain proteins, which contrasts with the number of genes that govern other second messengers such as cAMP^14,15^. This suggests that a complex c-di-GMP–based signaling network operates in diverse bacterial cells.

Multiple genes encoding DGCs and c-di-GMP PDEs are also found in the genomes of cyanobacteria, which are photoautotrophic organisms that perform oxygenic photosynthesis^16^. Although the prevalence of the c-di-GMP genes suggests that c-di-GMP has a crucial function in cyanobacterial physiology, the roles of c-di-GMP remains elusive^17^. Several studies have reported that c-di-GMP regulates phototactic motility^18,19^, biofilm formation, and cellular buoyancy^20^ of the cyanobacterium *Synechocystis*. However, no cellular target for c-di-GMP has yet been identified in cyanobacteria; thus the functions of c-di-GMP remain enigmatic.

We previously reported that c-di-GMP is a crucial inducer of cell aggregation of the thermophilic cyanobacterium *Thermosynechococcus vulcanus*^21^. The three cyanobacteriochrome-type photoreceptors SesA, SesB, and SesC, perceive light of the blue-to-green window and regulate cell aggregation. SesA is a blue light–activated DGC and acts as the major trigger of cell aggregation upon blue-light irradiation^22^. SesB exhibits PDE activity that is upregulated by teal light^23^ and GTP, and SesB represses cell aggregation under teal-green-light irradiation. SesC is a bifunctional photoreceptor, the DGC activity of which is induced by blue light and PDE activity is induced by green light, thereby enhancing the sensitivity of cells to incident light color. Thus, the coordinated action of SesA/B/C enables induction of cell aggregation specifically under blue light but not under teal-green light, and this is mediated by c-di-GMP signaling^21^.

In addition, the cellulose synthase Tll0007 has been identified as an essential component for cell aggregation^24^, with its PilZ domain being necessary for cell aggregation^22^. Thus, it is very likely that c-di-GMP activates the cellulose synthase function of Tll0007 similarly to its homolog BcsA proteins^1,2^, resulting in induction of the cellulose-dependent cell aggregation of *T. vulcanus*. However, the binding of c-di-GMP to the PilZ domain of Tll0007 lacks experimental verification. Moreover, the *Thermosynechococcus* genome contains a total of 10 genes encoding DGCs and c-di-GMP PDEs (Fig. S1). Although this complexity is much less than that of other cyanobacteria (e.g., 28 genes in *Synechocystis*), the relationship and interdependence of the c-di-GMP DGC and PDE proteins remain to be explored in *T. vulcanus*.

Here, we investigated the binding of c-di-GMP to Tll0007 and the kinetics of c-di-GMP production by SesA to assess the action of c-di-GMP in the signaling cascade. Furthermore, we performed systematic analyses of all the genes encoding DGCs and c-di-GMP PDEs to gain a clearer understanding of global c-di-GMP signaling network with respect to the regulation of cell aggregation. This study fosters a better understanding of the sophisticated light-regulated c-di-GMP signaling system in cyanobacteria. Various cyanobacteria are found in a sessile multicellular community in nature and thermophilic cyanobacteria *Thermosynechococcus* have been isolated from the top layer of microbial mats in hot springs^25–27^. The understanding of the molecular mechanisms underpinning cell aggregation and multicellular lifestyles will lead to integration of our knowledge of molecular physiology and ecology of cyanobacteria.

## Results

### The cellulose synthase Tll0007 binds c-di-GMP via its PilZ domain

**Figure 1:**
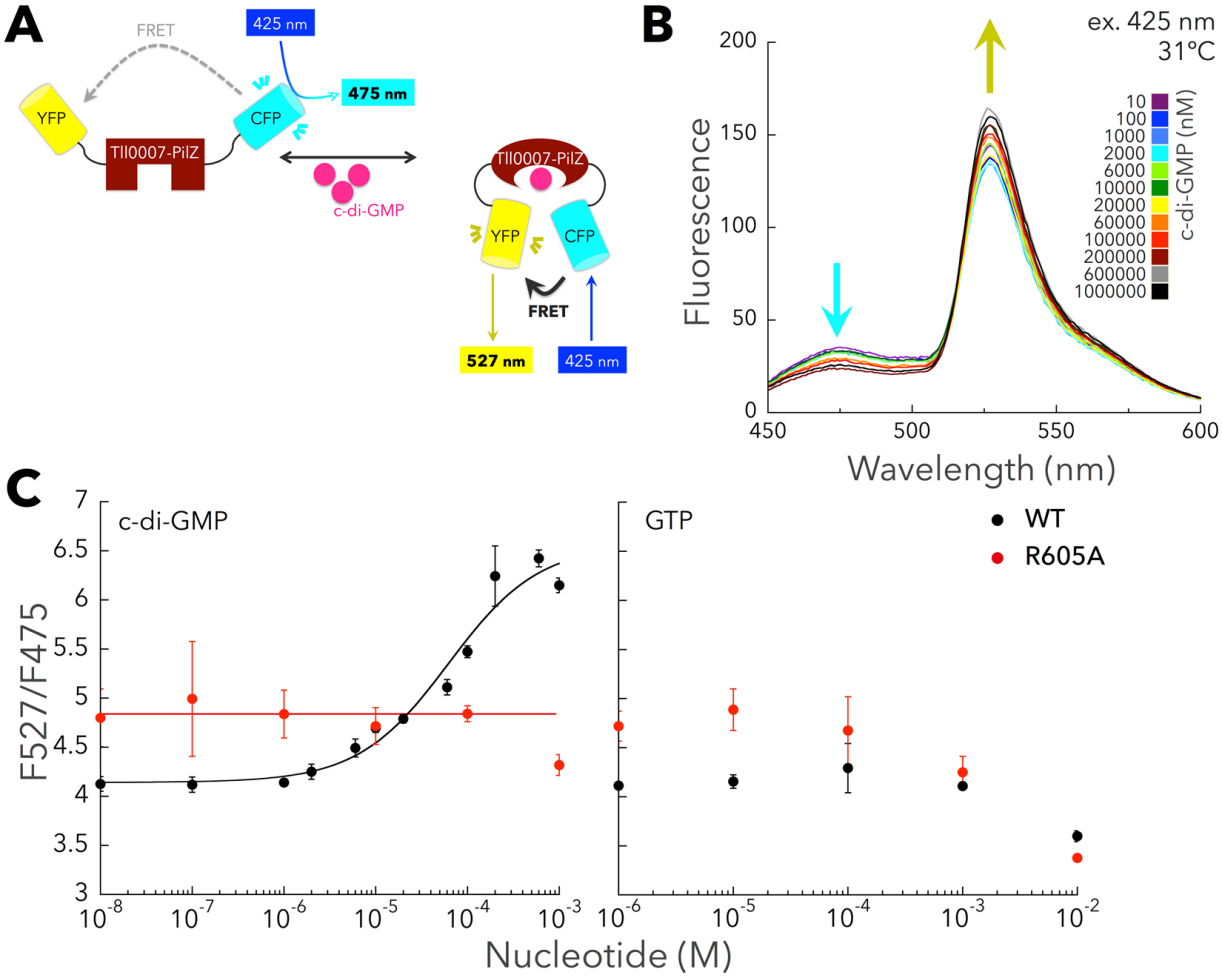
FRET-based biosensor assay for assessment of c-di-GMP binding to the PilZ domain of Tll0007. (**A**) Scheme illustrating how binding of c-di-GMP to a target protein domain (here, Tll0007-PilZ) leads to a change in FRET efficiency between the attached mCyPet (CFP) and mYPet (YFP). (**B**) Fluorescence emission spectra of the biosensor protein in the presence of the indicated concentrations of c-di-GMP. A representative of three independent experiments is shown. (**C**) Nucleotide concentration-response curve for FRET efficiency (YFP emission at 527 nm/CFP emission at 475 nm; F527/F475) for c-di-GMP (left) and GTP (right). Data represents the mean ± SD of at least three independent experiments. Black symbols, WT Tll0007; red symbols, R605A mutant.

We previously reported that both c-di-GMP and the PilZ domain of Tll0007 are essential for inducing cell aggregation of *T. vulcanus* at 31 °C^21,22^. However, experimental verification of c-di-GMP binding by the PilZ domain of Tll0007 was lacking. To assess c-di-GMP binding to Tll0007, in the present study we designed c-di-GMP biosensor proteins using a fluorescence resonance energy transfer (FRET)-based system by concatenating two fluorescent proteins (mCyPet and mYPet, which are monomerized variants of CFP and YFP, respectively) with the PilZ domain of Tll0007 in between (Fig. 1A), according to^28,29^. For each purified biosensor protein, the addition of c-di-GMP resulted in a corresponding increase in FRET efficiency, whereas addition of GTP did not (Fig. 1B). The c-di-GMP concentration-responsive curve revealed a dissociation constant (K_d_) of 63.9 ± 5.1 µM at 31 °C (Fig. 1C). Curve fitting revealed a Hill coefficient of 0.90 ± 0.14, indicating non-cooperative c-di-GMP binding.

The ^601^RXXXR^605^ motif in PilZ domains is necessary for binding c-di-GMP^30–32^. We thus created the mutant R605A and assessed the binding of c-di-GMP. R605A lacked the corresponding increase in FRET efficiency upon c-di-GMP addition (Fig. 1C), indicating that c-di-GMP binding depends on Arg605 in the RXXXR motif of Tll0007. R605A had somewhat higher FRET efficiency than the unmodified wild-type (WT) PilZ domain in the absence of added nucleotides, suggesting that R605A exists in a locked conformation that is slightly activated. These results suggested that Tll0007 binds c-di-GMP via its PilZ domain.

### The DGC activity of SesA exhibits product feedback inhibition

**Figure 2:**
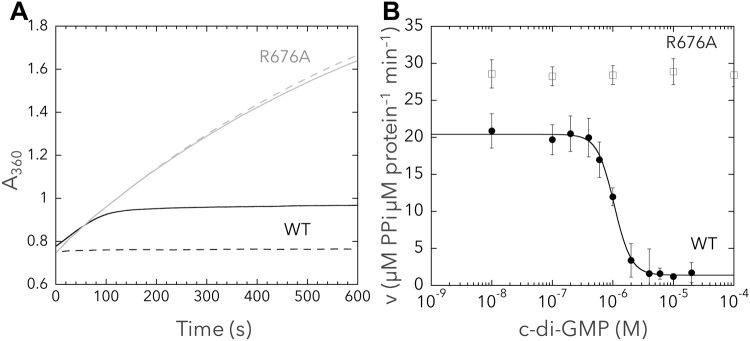
Pyrophosphate assay for assessing product feedback inhibition of SesA. (**A**) Mutant R676A or WT SesA (0.2 µM) was incubated at 31 °C in the absence (solid line) or presence of 10 µM c-di-GMP (broken line), and pyrophosphate production was measured by continuously monitoring absorbance at 360 nm. Representative results of three independent experiments are presented. Black lines, WT; grey lines, R676A. (**B**) C-di-GMP concentration-response curve for the initial velocity of the DGC reaction of SesA (µM PPi µM protein^−1^ min^−1^). A dose–response curve was fit for WT in KaleidaGraph software. PPi, pyrophosphate. Data represent the mean ± SD of three independent experiments. Solid circles, WT; open squares, R676A.

SesA is the major trigger of cell aggregation in *T. vulcanus* by producing c-di-GMP under blue-light irradiation^21,22^. The DGC activities of many GGDEF proteins are negatively regulated by their product, i.e., c-di-GMP^3,33^. To assess any potential product feedback inhibition of SesA, we measured the initial rate of the DGC reaction in the presence of various concentrations of c-di-GMP using a pyrophosphate assay kit, which monitors production of pyrophosphate—the byproduct of the DGC reaction. The purified protein of SesA was pre-irradiated with blue light to retain it in its active form during the assay. In the absence of c-di-GMP, WT SesA showed high initial pyrophosphate production activity, followed by a gradual decrease in production with increasing c-di-GMP concentration, which eventually stopped after ~100 s (at approximately 5 µM c-di-GMP) (Fig. 2A). Conversely, in the presence of 10 µM c-di-GMP, the DGC activity of WT SesA was strongly inhibited. These results indicated that the DGC activity of SesA is subject to product feedback inhibition.

The concentration-responsive curve for the DGC activity in the presence of c-di-GMP indicated an IC50 value (concentration for half-maximal inhibition) of 1.07 ± 0.13 µM at 31 °C (Fig. 2B). This affinity of c-di-GMP for the product inhibition site of SesA is comparable to values reported for other DGCs such as DgcA (IC50 = 0.96 µM) and PleD (IC50 = 5.8 µM)^33^. The DGC activity of SesA in the absence of c-di-GMP was 20.5 ± 2.1 µmol pyrophosphate µmol protein^−1^ min^−1^; i.e., a turnover rate (k_cat_) ~10 min^−1^. Curve fitting also indicated a Hill coefficient of 3.0 ± 0.3, implying high positive cooperativity of c-di-GMP binding to SesA.

Upon product feedback inhibition of DGCs, c-di-GMP binds to the allosteric product inhibition site (I-site), namely the RXXD motif^33^, which is located near the active-site within the GGDEF domain^34–36^. We used the SesA mutant R676A (within ^676^RXXD) to assess product inhibition. This mutant showed high pyrophosphate production activity regardless of the presence of 10 µM c-di-GMP, with the activity being sustained during the assay for 600 s (Fig. 2A). In fact, R676A maintained its high DGC activity even in the presence of 100 µM c-di-GMP (Fig. 2B), indicating that the RXXD motif is necessary for product inhibition of SesA. R676A had higher DGC activity than WT SesA even in the absence of c-di-GMP, suggesting that the R676A mutation alone stabilized the active enzyme conformation. The protein oligomerization of SesA may have been altered by the R676A mutation, as reported for the GGDEF domain of *Thermotoga maritima* TM1788^37,38^. Taken together, these results demonstrate that SesA is subject to product feedback inhibition by c-di-GMP with relatively high affinity.

### Transcriptional regulation of the genes encoding DGC and PDEs

Given the above results, the amount of c-di-GMP produced by SesA (IC50 ~ 1 µM) may be insufficient to activate Tll0007 (K_d_ ~ 64 µM). The gap between these values might imply that another step is necessary to amplify the c-di-GMP signal from SesA toward Tll0007, such as c-di-GMP–dependent transcriptional or enzymatic regulation of DGC and PDEs. In addition to SesA/B/C, seven other genes encode GGDEF/EAL/HD-GYP–domain proteins in *Thermosynechococcus* (Fig. S1). An amino acid sequence alignment of the GGDEF domains revealed that the residues necessary for DGC activity^39^ are conserved in all GGDEF domains except for that in SesB (Fig. S2), which lacks DGC activity^21^. The RXXD motif of the GGDEF domain is conserved in SesA, SesC, Tlr1210, Tlr0627, Tll1049, and Tll1859, suggesting that some of these proteins may be subject to product inhibition. All the EAL domains in *Thermosynechococcus* appeared to be active based on an amino acid sequence alignment (Fig. S3)^39,40^. Thus, the *in silico* analyses did not identify any genes encoding enzymatically inactive GGDEF/EAL/HD-GYP domains.

We assessed the transcriptional regulation of the 10 genes by real-time qPCR analysis. Total RNA was prepared from (1) cells irradiated with white light at 45 °C (standard condition), (2) cells irradiated with blue light at 31 °C (cell aggregation-ON condition), and (3) cells irradiated with teal-green light at 31 °C (cell aggregation-OFF condition). The relative expression of each gene was evaluated compared with the standard condition (Table 1). All the 10 genes were transcribed to a greater degree at 31 °C than at 45 °C, suggesting that a relatively low temperature leads to activation of the whole c-di-GMP signaling network. None of the genes were differentially expressed significantly between the cells under blue light or teal-green light. We previously reported that blue light- and green light-regulated production and degradation of c-di-GMP control the cell-aggregation-ON and –OFF regulation, respectively^21^. These results suggested that the c-di-GMP levels set by SesA/B/C in response to light color did not induce transcriptional change of genes encoding GGDEF/EAL/HD-GYP domain proteins; thus, post-transcriptional control of the GGDEF/EAL/HD-GYP domain proteins is crucial for determining whether cell aggregation is induced or repressed at relatively low temperatures.

**Table 1:** Results for the real-time qPCR analysis of 10 genes encoding DGCs and PDEs.

Gene	Relative expression (vs. irradiation with white light at 45 °C)	
	Blue light at 31 °C	Teal-green light at 31 °C
*sesA*	5.4 ± 4.7	6.5 ± 3.3
*sesB*	2.5 ± 0.7	2.1 ± 0.3
*sesC*	4.9 ± 2.8	7.9 ± 3.9
*tlr1210*	45 ± 43	89 ± 65
*tlr1158*	3.2 ± 1.4	6.1 ± 4.7
*tll1049*	4.2 ± 0.6	4.5 ± 1.8
*tll1859*	4.1 ± 1.3	6.0 ± 1.3
*tll0627*	4.1 ± 1.5	6.2 ± 4.3
*tlr1612*	5.6 ± 3.5	5.9 ± 2.7
*tlr0485*	7.0 ± 3.1	7.9 ± 3.1

The mRNA level for each gene encoding a GGDEF/EAL/HD-GYP domain proteins was determined with total RNA samples from (1) cells irradiated with white light at 45°C (standard condition), (2) cells irradiated with blue light at 31°C (cell aggregation-ON condition), and (3) cells irradiated with teal-green light at 31 °C (cell aggregation-OFF condition). The relative expression of each target gene was determined by normalization to the internal control gene *rnpB*. The results represent the mean ± SD of values from three independent RNA preparations.

### Involvement of other DGC and PDEs in the regulation of cell aggregation

**Figure 3:**
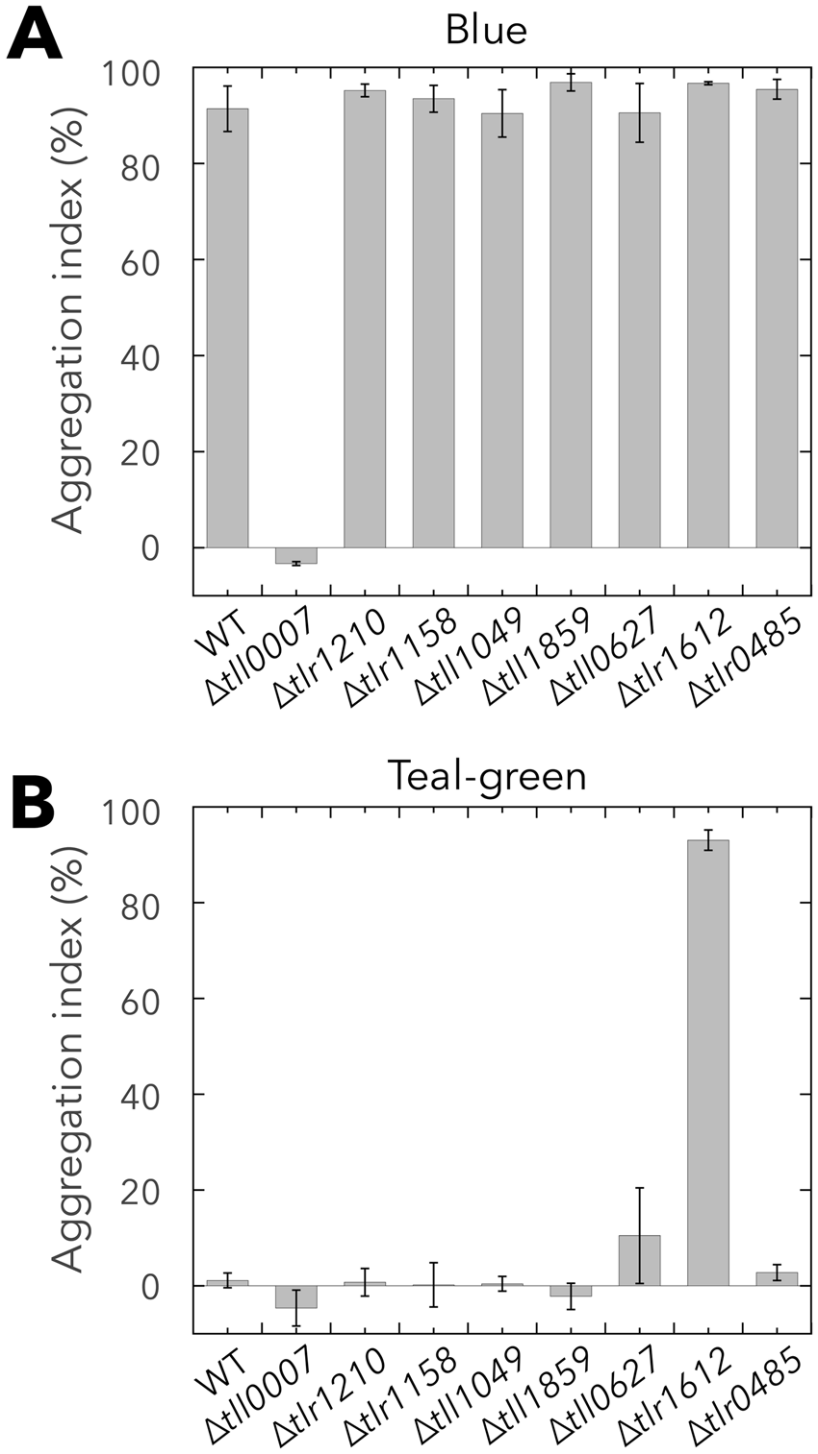
Systematic assessment of the cell aggregation of single DGC/PDE-encoding gene disruption mutants. Aggregation index values for WT and single gene–disrupted mutants are shown. Cells were cultured at 31 °C for 48 h under blue light (**A**) or teal-green light (**B**). Data represent the mean ± SD of at least three biological replicates.

We created gene-disruption mutants of the remaining seven genes (*tlr1210*, *tlr1158*, *tll1049*, *tll1859*, *tll0627*, *tlr1612*, and *tlr0485*) of *T. vulcanus* and analyzed the aggregation of cells irradiated with blue or teal-green light irradiation at 31 °C (Fig. 3). The phenotypes of the other three mutants (*sesA*, *sesB*, and *sesC*) have already been reported^21^. Under blue light, WT cells underwent strong cell aggregation whereas ∆*tll0007* mutant cells did not aggregate, in agreement with our previous reports^22,24^. None of the seven gene-disruption mutants displayed altered cell aggregation, indicating that no other DGC besides SesA is essential for cell aggregation. Under teal-green light, ∆*tlr1612* cells underwent strong aggregation that was comparable with the aggregation observed under blue light, whereas the WT and other mutants did not aggregate. ∆*sesB* cells also underwent some aggregation under teal-green light, although the aggregation index value was much lower (~20%)^21^. These results suggested that Tlr1612 is the primary protein that represses the aggregation of cells irradiated with teal-green-light.

### Tlr1612 is epistatic to SesA for the regulation of cell aggregation

**Figure 4:**
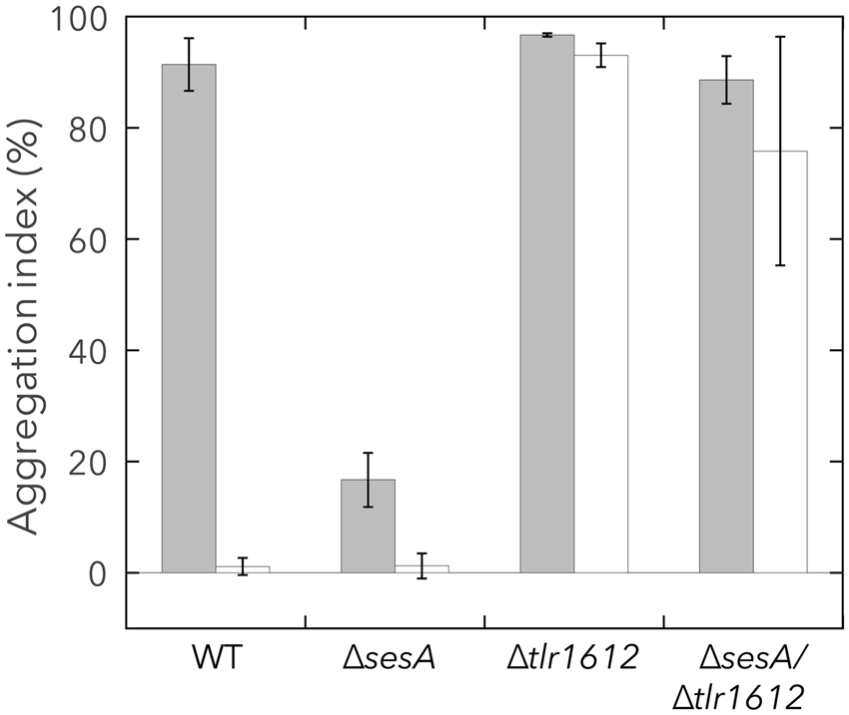
Epistasis analysis of *sesA* and *tlr1612*. Aggregation index values for WT and gene-disrupted mutants are shown. Cells were cultured at 31 °C for 48 h under blue light (filled bar) or teal-green light (open bar). Data represent the mean ± SD of at least three biological replicates. The data for WT and ∆*tlr1612* are duplicated from Fig. 3 for comparison.

∆*tlr1612* cells underwent strong aggregation under blue or teal-green light (Fig. 3), whereas ∆*sesA* cells showed little aggregation under either condition^21,22^. We therefore examined the possible epistasis of *sesA* and *tlr1612* by creating the ∆*sesA*/∆*tlr1612* double mutant. ∆*sesA*/∆*tlr1612* cells underwent strong aggregation under both light conditions, similar to ∆*tlr1612* (Fig. 4), indicating that *tlr1612* is epistatic to SesA for the regulation of cell aggregation.

## Discussion

We investigated the c-di-GMP signaling network that regulates light-dependent cell aggregation in *T. vulcanus*. The cooperating cyanobacteriochrome photoreceptors SesA/B/C perceive blue-to-green light as an input to regulate c-di-GMP signaling^21^. However, it has remained elusive how other c-di-GMP signaling proteins function. The current study has refined our previous work and deepened our understanding of the sophisticated light-dependent c-di-GMP signaling network.

Tll0007 is the first cyanobacterial c-di-GMP receptor to be experimentally validated, corroborating the existence of c-di-GMP signaling in cyanobacteria. The affinity of the PilZ domain of Tll0007 for c-di-GMP (K_d_ = 63.9 ± 5.1 µM) (Fig. 1) was relatively low compared with other c-di-GMP receptors such as the *Vibrio cholerae* PlzD (K_d_ = 0.2 µM) and VpsT (K_d_ = 3.2 µM) and *Pseudomonas aeruginosa* Alg44 (K_d_ = 12.7 µM)^12,28,41^. Using our FRET biosensor assay, the full-length YcgR of *Escherichia coli* yielded an affinity of 0.42 ± 0.08 µM (Fig. S4), which is consistent with a previously reported value (K_d_ = 0.84 ± 0.16 µM)^9^, underscoring the reliability of our assay. We cannot exclude the possibility that the affinity of full-length Tll0007 for c-di-GMP is higher *in vivo*. However, *Salmonella* Typhimurium BcsA, a homolog of Tll0007, was found to have higher affinity (K_d_ = 8.4 µM) than Tll0007 even with a similar construct and assay^28^. These results strongly suggest that a relatively high cellular concentration of c-di-GMP may be necessary to activate Tll0007.

The Hill coefficient for Tll0007 was 0.90 ± 0.14 (Fig. 1), which contrasts with other PilZ domains that generally show positive cooperativity for c-di-GMP binding^28,42^. PilZ domains usually bind the c-di-GMP dimer^2,31,32,43^, although binding of the c-di-GMP monomer has also been reported^42^. The lack of cooperativity of c-di-GMP binding to Tll0007 suggests that Tll0007 can bind the c-di-GMP monomer and does not specifically recognize the c-di-GMP dimer. Notably, the binding of the c-di-GMP dimer to the PilZ domain protein Alg44 could induce functional output, whereas binding of the c-di-GMP monomer could not, indicating that a specific form of c-di-GMP is necessary for protein activation^31^. The high cooperativity that has been demonstrated for most PilZ domains appears to reflect the preference for binding the c-di-GMP dimer. Conversely, Tll0007 might be activated by the c-di-GMP monomer.

SesA is the major trigger of cell aggregation for *T. vulcanus*^21^, and we found that SesA is subject to the product feedback inhibition (Fig. 2). The high cooperativity of product inhibition permits SesA to respond in an all-or-none fashion over a narrow range of c-di-GMP concentration^44^. The DGC activity of SesA (k_cat_ ~10 min^−1^) is relatively high compared with known DGCs such as TM1788(R158A) (k_cat_ ~2.6 min^−1^)^37^ and YdeH/DgcZ (k_cat_ ~1.6 min^−1^)^45^, and comparable with Cph2 (k_cat_ ~10.6 min^−1^)^18^. Such high DGC activity is likely important for SesA to rapidly produce sufficient c-di-GMP for the induction of cell aggregation upon blue-light exposure. Notably, *Synechocystis* cells over-expressing SesA (R676A) were inviable under white-light irradiation, whereas these cells grew normally when irradiated with both green and red light. This finding suggests that the high and unlimited DGC activity of SesA (R676A), when activated by blue light, is toxic to the *Synechocystis* cells, probably owing to exhaustion of GTP substrate and/or overactivation of c-di-GMP signaling. Thus, it is logical that product feedback inhibition is necessary to control the high DGC activity of SesA. Product feedback inhibition is assumed to be common for GGDEF domain proteins and has been intensively investigated *in vitro*^33–35,46^. However, the physiological importance of DGC product inhibition remains an unresolved issue.

**Figure 5:**
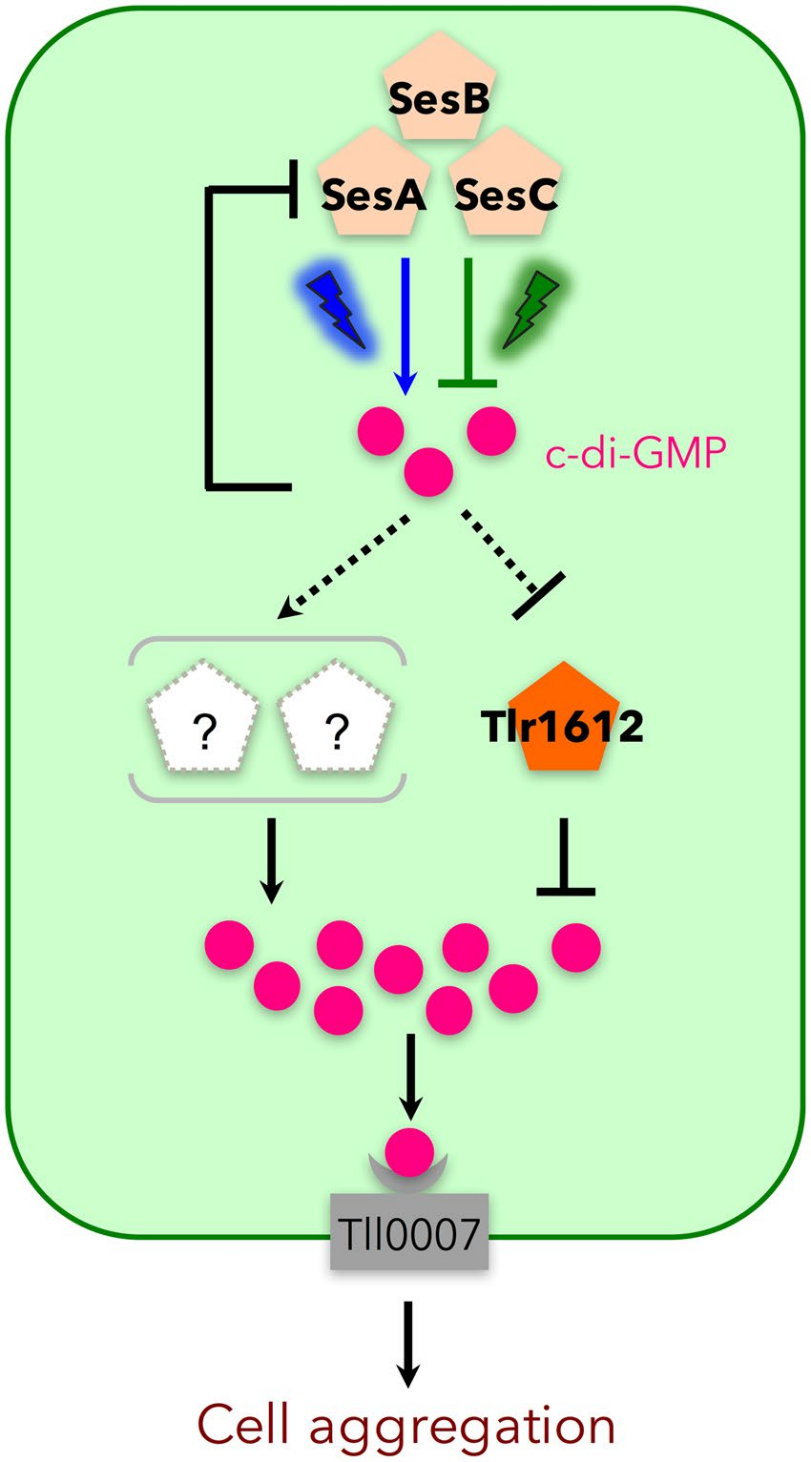
Refined model for light wavelength-dependent c-di-GMP signaling for *Thermosynechococcus vulcanus* cell aggregation. Blue light regulates the activities of SesA/B/C concertedly to increase c-di-GMP. A relatively low c-di-GMP concentration is maintained via strict product feedback inhibition of the DGC activity of the major trigger, SesA. The c-di-GMP signal may lead to an increased cellular concentration of c-di-GMP possibly via post-transcriptional regulation of Tlr1612 and/or other DGC/PDE proteins. At higher concentrations, c-di-GMP binds to and activates the cellulose synthase Tll0007, leading to cell aggregation.

Our study identified Tlr1612 as the crucial protein to repress cell aggregation under teal-green light (Fig. 3B). The expression of *tlr1612* did not differ between the blue-light and teal-green-light conditions (Table 1), excluding the possibility that transcriptional regulation underlies the activation of *tlr1612*. However, *tlr1612* was found to be epistatic to *sesA* (Fig. 4), indicating that Tlr1612 acts downstream of SesA in the c-di-GMP signaling network. Furthermore, our biochemical studies demonstrated that SesA-produced c-di-GMP levels barely exceeded ~5 µM owing to product inhibition (Fig. 2), with this level being far lower than the K_d_ value for binding to Tll0007 (Fig. 1). However, no other single DGC was responsible for cell aggregation under blue light, whereas *tlr1612* was essential for suppression of cell aggregation under teal-green light (Fig. 3, and see^21^ for ∆*sesB* and ∆*sesC*). Although the predicted Tlr1612 protein harbors both GGDEF and EAL domains, it probably serves as the major PDE that suppresses cell aggregation under teal-green light and likely under blue light as well, according to our gene expression analysis. Nevertheless, SesA was able to trigger cell aggregation under blue light, indicative of a certain mechanism(s) that leads to an upshift of c-di-GMP level sufficient for the low-affinity site of Tll0007. At present, it is not known whether such an upshift could be achieved by SesA-dependent downregulation of Tlr1612 PDE, SesA-dependent upregulation of Tlr1612 DGC, or SesA-dependent upregulation of two or more yet unidentified DGCs including SesC (as upregulation of any single DGC except SesA was excluded by our gene disruption experiments). These scenarios are summarized in Fig. 5.

Notably, disruption of all the three photoreceptors (SesA/B/C) still allowed some cell aggregation (the aggregation index was 40–70% for all the tested wavelengths^21^). This observation implies that certain levels of c-di-GMP are maintained during normal phototrophic growth owing to production by one or more DGC(s), even though the possibly strong PDE activity of Tlr1612 is also functioning. There are many indications that multiple DGCs and PDEs are often spatially differentiated in prokaryotic cells, in addition to the well-known temporal regulations^4,47^. The possible PDE activity of Tlr1612 may also serve as an insulator for other, potentially unrelated, effects of c-di-GMP signaling, which are not shown in Fig. 5. To reconcile the ambiguity of our model, the biochemical properties of Tlr1612 and of other DGC proteins must at least be ascertained. A somewhat similar two-step c-di-GMP signaling system has been reported in *Salmonella*, wherein the “first” c-di-GMP pool activates the transcription of the gene encoding the DGC protein AdrA, which produces higher levels of the “second” c-di-GMP pool that directly activates the cellulose synthase activity^48,49^. Such transcriptional regulation is unlikely in *Thermosynechococcus* as no gene exhibited a difference in transcript levels between the cell aggregation-ON and -OFF conditions (Table 1). Thus, post-transcriptional control will surely be of importance in the c-di-GMP signaling network of *Thermosynechococcus*, as also proposed for *E. coli*^50^.

Besides SesA/B/C and Tlr1612, six other genes encode GGDEF/EAL/HD-GYP domain proteins in *Thermosynechococcus*, although their functions remain completely unknown. Disruption of each of these six genes did not affect cell aggregation at 31 °C (Fig. 3), suggesting that these genes may have redundant roles in cell aggregation or be involved in other cellular responses—potentially regulated by c-di-GMP. All of the c-di-GMP signaling genes, especially *tlr1210*, were expressed to a greater degree at 31 °C compared with 45 °C (Table 1), suggesting that the overall c-di-GMP signaling governs various cellular responses especially at relatively low temperatures. A candidate for a c-di-GMP–regulated cellular responses is phototactic motility; indeed, c-di-GMP regulates phototaxis in the cyanobacterium *Synechocystis*^18,19^. The PilB ATPase, which drives the extension of type IV pili^51,52^, is a likely universal c-di-GMP–binding protein^10^. Future work will address how each GGDEF/EAL/HD-GYP protein employs c-di-GMP to orchestrate cellular responses in cyanobacteria. The protein thermostability and the relatively small number of c-di-GMP genes of *Thermosynechococcus* suggest the potential for further detailed characterization of every protein in the sophisticated c-di-GMP signaling network.

## Methods

### Construction of plasmids and mutants

Primers used are listed in Table S1. Plasmids were constructed using the In-Fusion System (TaKaRa). For constructing FRET biosensors, fluorescent proteins were cloned from pCyPet-His and pYPet-His (Addgene). We created monomerized variants of CyPet (mCyPet) and YPet (mYPet) by introducing A206K^29,53^. The DNA encoding the chimeric protein consisting of mCyPet, Tll0007-PilZ (or the full-length YcgR of *E. coli*), and mYPet was cloned into pET28V, in which the protease recognition site in the original pET28a plasmid (Novagen), for removal of the N-terminal His tag, was replaced with one for tobacco etch virus (TEV) protease. Site-directed mutagenesis was performed using PrimeSTAR Max Basal Mutagenesis kit reagents (TaKaRa). The vector pTCH2031V was used to overexpress SesA in *Synechocystis* sp. PCC 6803^21^. The protease recognition site for removal of the N-terminal His tag was replaced with one for TEV protease, which could be used for future purification. The *E. coli rrnB* terminator sequence was inserted after the C-terminus of the protein-coding region incorporated into pTCH2031V.

For disruption of *tlr1210*, *tlr1158*, *tll1049*, *tll1859*, *tll0627*, and *tlr1612* in *T. vulcanus*, a spectinomycin/streptomycin-resistance cassette was inserted into the start codon of each gene. For disruption of *tlr0485*, the majority of the open reading frame was replaced by a chloramphenicol resistance-cassette.

### Protein purification

*E. coli* and *Synechocystis* protein-expressing cells were harvested by centrifugation at 4450 × *g* for 10 min, suspended in 50 mM Hepes·NaOH (pH 7.5) containing 300 mM NaCl, 10% (wt/vol) glycerol, 30 mM imidazole, and 0.5 mM Tris(2-carboxylethyl)phosphine, and then frozen at −80 °C. After thawing, cells were subjected to three rounds of disruption using a French press (5501-M; Ohtake) at 1,500 kg·cm^−2^. Each cyanobacterial cell homogenate was centrifuged at 12,000 × *g* for 10 min and then at 194,100 × *g* for 30 min. Each *E. coli* homogenate was centrifuged at 194,100 × *g* for 30 min. Each supernatant was filtered through a 0.8-µm cellulose acetate filter and then loaded onto a nickel-affinity His-Trap chelating column (GE Healthcare). Proteins were eluted with a linear gradient of 30–430 mM imidazole in 20 mM Hepes·NaOH (pH 7.5), 300 mM NaCl, 10% (wt/vol) glycerol, and 0.5 mM Tris(2-carboxylethyl)phosphine. Ethylenediaminetetraacetic acid (1 mM) was added to the pooled peak fractions, which were then dialyzed against 20 mM Hepes·NaOH (pH 7.5), 300 mM NaCl, 10% (wt/vol) glycerol, and 1 mM dithiothreitol (DTT). For purification of FRET-based biosensor proteins, the reducing agents (Tris(2-carboxylethyl)phosphine and DTT) were omitted.

### Fluorescence spectroscopy

Fluorescence spectra were recorded at 31 °C using a RF-5300PC spectrofluorometer (Shimadzu, excitation at 425 nm, 5 nm excitation and 3 nm emission slit widths, emission scan from 450 to 600 nm) using 50 nM of an individual protein in 20 mM Hepes·NaOH (pH 7.5), 100 mM NaCl, and 10% (wt/vol) glycerol. Binding affinity was determined by adding increasing concentrations of c-di-GMP (10 nM to 1 mM) or GTP (1 µM to 10 mM) and measuring the resulting YFP/CFP emission ratio (emission at 527 nm/emission at 475 nm). Curve fitting was performed using the program KaleidaGraph (Synergy Software).

### Kinetic measurements of DGC activity

The amount of inorganic pyrophosphate, a by-product of c-di-GMP synthesis, was monitored at 360 nm with a UV-2600PC spectrophotometer using EnzChek pyrophosphate assay kit reagents (Invitrogen)^22,54^. The kit includes inorganic pyrophosphatase, which catalyzes conversion of inorganic pyrophosphate into 2 equivalents of inorganic phosphate. In the presence of inorganic phosphate, the substrate 2-amino-6-mercapto-7-methylpurine ribonucleoside was enzymatically converted to ribose 1-phosphate and 2-amino-6-mercapto-7-methylpurine, which absorbs 360-nm light. Each reaction contained 50 mM Tris-HCl, pH 7.5, 20 mM MgCl_2_, and 0.2 µM SesA in the presence of a varying amount of c-di-GMP. The reaction was initiated by the addition of 100 µM GTP, incubated at 31 °C, and monitored for 600 s.

### Cyanobacterial strains and culture conditions

The *T. vulcanus* strain RKN (equivalent to National Institute for Environmental Studies 2134) that shows positive phototaxis was cultured at 45 °C in BG11 medium^55^. Culture density was monitored at 730 nm. Transformations of *T. vulcanus* were performed according to^56^. The antibiotic concentration in BG11 medium for selection of transformants was 5 µg·mL^−1^ chloramphenicol, 80 µg·mL^−1^ kanamycin, or 10 µg·mL^−1^ spectinomycin plus 5 µg·mL^−1^ streptomycin. PCR was used to confirm the complete segregation of the mutant allele (i.e., the WT loci were replaced with the mutant loci in all of the multiple copies of the cyanobacterial chromosome).

### Real-time qPCR assay

Total RNA was extracted from *T. vulcanus* cells using the Qiagen RNeasy RNA purification kit with RNA Protect Bacteria Reagent (Qiagen). Cells were grown (1) under irradiation of a white-light fluorescent lamp (35 µmol photon· m^−2^·s^−1^) at 45 °C (standard condition), (2) under blue light (λ_max_ = 448 nm; 5 µmol photon· m^−2^·s^−1^; Valore Corp.) with photosynthetic red light (λ_max_ = 634 nm; 30 µmol photon· m^−2^·s^−1^; Valore Corp.) at 31 °C (cell aggregation-ON condition), or (3) under teal-green light (λ_max_ = 507 nm; 5 µmol photon· m^−2^·s^−1^; Valore Corp.) with the red light at 31 °C (cell aggregation-OFF condition) for 48 h^21^. RNA integrity was checked by agarose gel electrophoresis. cDNA was synthesized via reverse transcription of RNA (0.8 μg) from each sample using the PrimeScript RT reagent Kit with gDNA Eraser (TaKaRa). Real-time qPCR was performed on a Takara Thermal Cycler Dice. Each 20-μL reaction contained 1 μL of cDNA, 300 nM of each primer (Table S2), and 10 μL THUNDERBIRD SYBR qPCR Mix (Toyobo). Samples were denatured initially by heating at 95 °C for 1 min, followed by a 40-cycle amplification and quantification program (95 °C for 15 s and 60 °C for 30 s). Melting-curve analysis was conducted to ensure amplification of a single product. The amplification efficiency of each primer pair was determined by running three-fold serial dilutions (seven dilution series). A standard curve was generated by plotting the cycle threshold (C_t_) value determined using the second derivative maximum method against the log of the dilution factor. Gene expression was normalized to that of *rnpB*^57^.

### Cell aggregation assay

Cultures of *T. vulcanus* WT and its disrupted mutants grown at 45 °C (OD_730_ 0.5–2) were diluted to yield a culture OD_730_ of 0.2. These samples were then incubated at 31 °C for 48 h under photosynthetic red light along with blue or teal-green light. The results of the cellulose-dependent cell-aggregation assay, described as follows, were reported as an aggregation index (%)^24^. Briefly, after the irradiation period, cell suspensions were thoroughly mixed and aliquots transferred to cuvettes. The samples were held at room temperature in the dark for 30 min, during which time most of the aggregated cells precipitated. Then, the OD_730_ of each sample was measured (denoted OD_NA_; i.e., OD_730_ of non-aggregated cells remaining in the culture medium). Next, cellulase (12.5 U·mL^−1^; Worthington Biochemical) was added to each cuvette sample, which was then incubated for 30 min at 37 °C to completely disperse the aggregated cells. The OD_730_ of each sample (denoted OD_total_) was then measured. The aggregation index (%) was defined as ((OD_total_ − OD_NA_)/OD_total_) × 100.

## Electronic supplementary material

Supplementary Information

## References

[1] Ross P (1987). Regulation of cellulose synthesis in *Acetobacter xylinum* by cyclic diguanylic acid. Nature.

[2] Morgan JL, McNamara JT, Zimmer J (2014). Mechanism of activation of bacterial cellulose synthase by cyclic di-GMP. Nat Struct Mol Biol.

[3] Römling U, Galperin MY, Gomelsky M (2013). Cyclic di-GMP: the first 25 years of a universal bacterial second messenger. Microbiol Mol Biol Rev.

[4] Jenal U, Reinders A, Lori C (2017). Cyclic di-GMP: second messenger extraordinaire. Nat Rev Microbiol.

[5] Paul R (2004). Cell cycle-dependent dynamic localization of a bacterial response regulator with a novel di-guanylate cyclase output domain. Genes Dev.

[6] Christen M, Christen B, Folcher M, Schauerte A, Jenal U (2005). Identification and characterization of a cyclic di-GMP-specific phosphodiesterase and its allosteric control by GTP. J Biol Chem.

[7] Ryan RP (2006). Cell-cell signaling in *Xanthomonas campestris* involves an HD-GYP domain protein that functions in cyclic di-GMP turnover. Proc Natl Acad Sci USA.

[8] Amikam D, Galperin MY (2006). PilZ domain is part of the bacterial c-di-GMP binding protein. Bioinformatics.

[9] Ryjenkov DA, Simm R, Römling U, Gomelsky M (2006). The PilZ domain is a receptor for the second messenger c-di-GMP: the PilZ domain protein YcgR controls motility in enterobacteria. J Biol Chem.

[10] Wang YC (2016). Nucleotide binding by the widespread high-affinity cyclic di-GMP receptor MshEN domain. Nature communications.

[11] Hickman JW, Harwood CS (2008). Identification of FleQ from *Pseudomonas aeruginosa* as a c-di-GMP-responsive transcription factor. Mol Microbiol.

[12] Chou S-H, Galperin MY (2016). Diversity of c-di-GMP-binding proteins and mechanisms. J Bacteriol.

[13] Sudarsan N (2008). Riboswitches in eubacteria sense the second messenger cyclic di-GMP. Science.

[14] Massie JP (2012). Quantification of high-specificity cyclic diguanylate signaling. Proc Natl Acad Sci USA.

[15] Hengge R (2009). Principles of c-di-GMP signalling in bacteria. Nat Rev Microbiol.

[16] Agostoni M, Koestler BJ, Waters CM, Williams BL, Montgomery BL (2013). Occurrence of cyclic di-GMP-modulating output domains in cyanobacteria: an illuminating perspective. mBio.

[17] Agostoni M, Montgomery BL (2014). Survival strategies in the aquatic and terrestrial world: the impact of second messengers on cyanobacterial processes. Life.

[18] Savakis P (2012). Light-induced alteration of c-di-GMP level controls motility of *Synechocystis* sp. PCC 6803. Mol Microbiol.

[19] Angerer V (2017). The protein Slr1143 is an active diguanylate cyclase in *Synechocystis* sp. PCC 6803 and interacts with the photoreceptor Cph2. Microbiology.

[20] Agostoni M, Waters CM, Montgomery BL (2016). Regulation of biofilm formation and cellular buoyancy through modulating intracellular cyclic di-GMP levels in engineered cyanobacteria. Biotechnol Bioeng.

[21] Enomoto G, Ni Ni W, Narikawa R, Ikeuchi M (2015). Three cyanobacteriochromes work together to form a light color-sensitive input system for c-di-GMP signaling of cell aggregation. Proc Natl Acad Sci USA.

[22] Enomoto G (2014). Cyanobacteriochrome SesA is a diguanylate cyclase that induces cell aggregation in. Thermosynechococcus. J Biol Chem.

[23] Enomoto G, Hirose Y, Narikawa R, Ikeuchi M (2012). Thiol-based photocycle of the blue and teal light-sensing cyanobacteriochrome Tlr1999. Biochemistry.

[24] Kawano Y (2011). Cellulose accumulation and a cellulose synthase gene are responsible for cell aggregation in the cyanobacterium *Thermosynechococcus vulcanus* RKN. Plant Cell Physiol.

[25] Stal LJ (2017). Gregarious cyanobacteria. Environ Microbiol.

[26] Koike, H. & Inoue, Y. Preparation of oxygen-evolving photosystem II particles from a thermophilic blue-green alga. In: *The Oxygen Evolving System of Photosynthesis* (eds). Academic Press (1983).

[27] Stolyar S, *et al*. Genome sequence of the thermophilic cyanobacterium *Thermosynechococcus* sp. strain NK55a. *Genome announcements* **2** (2014). 10.1128/genomeA.01060-13 PMC3907722 24482507

[28] Pultz IS (2012). The response threshold of *Salmonella* PilZ domain proteins is determined by their binding affinities for c-di-GMP. Mol Microbiol.

[29] Christen M (2010). Asymmetrical distribution of the second messenger c-di-GMP upon bacterial cell division. Science.

[30] Christen M (2007). DgrA is a member of a new family of cyclic diguanosine monophosphate receptors and controls flagellar motor function in *Caulobacter crescentus*. Proc Natl Acad Sci USA.

[31] Whitney JC (2015). Dimeric c-di-GMP is required for post-translational regulation of alginate production in *Pseudomonas aeruginosa*. J Biol Chem.

[32] Ko J (2010). Structure of PP4397 reveals the molecular basis for different c-di-GMP binding modes by PilZ domain proteins. J Mol Biol.

[33] Christen B (2006). Allosteric control of cyclic di-GMP signaling. J Biol Chem.

[34] Chan C (2004). Structural basis of activity and allosteric control of diguanylate cyclase. Proc Natl Acad Sci USA.

[35] De N, Navarro MV, Raghavan RV, Sondermann H (2009). Determinants for the activation and autoinhibition of the diguanylate cyclase response regulator WspR. J Mol Biol.

[36] Wassmann P (2007). Structure of BeF_3_^−^modified response regulator PleD: implications for diguanylate cyclase activation, catalysis, and feedback inhibition. Structure.

[37] Rao F (2009). Enzymatic synthesis of c-di-GMP using a thermophilic diguanylate cyclase. Anal Biochem.

[38] Deepthi A, Liew CW, Liang ZX, Swaminathan K, Lescar J (2014). Structure of a diguanylate cyclase from Thermotoga maritima: insights into activation, feedback inhibition and thermostability. PloS one.

[39] Schirmer T, Jenal U (2009). Structural and mechanistic determinants of c-di-GMP signalling. Nat Rev Microbiol.

[40] Sundriyal A (2014). Inherent regulation of EAL domain-catalyzed hydrolysis of second messenger cyclic di-GMP. J Biol Chem.

[41] Krasteva PV (2010). *Vibrio cholerae* VpsT regulates matrix production and motility by directly sensing cyclic di-GMP. Science.

[42] Benach J (2007). The structural basis of cyclic diguanylate signal transduction by PilZ domains. EMBO J.

[43] Habazettl, J., Allan, M. G., Jenal, U. & Grzesiek, S. Solution structure of the PilZ domain protein PA4608 complex with cyclic di-GMP identifies charge clustering as molecular readout. *J Biol Chem* **286**, 14304–14314 (2011). 10.1074/jbc.M110.209007 PMC3077631 21310957

[44] Oliveira MC (2015). Cooperative substrate binding by a diguanylate cyclase. J Mol Biol.

[45] Boehm A (2009). Second messenger signalling governs *Escherichia coli* biofilm induction upon ribosomal stress. Mol Microbiol.

[46] Whiteley CG, Lee DJ (2015). Bacterial diguanylate cyclases: structure, function and mechanism in exopolysaccharide biofilm development. Biotechnol Adv.

[47] Sarenko O, *et al*. More than enzymes that make or break cyclic di-GMP-local signaling in the interactome of GGDEF/EAL domain proteins of *Escherichia coli*. *mBio* **8** (2017). 10.1128/mBio.01639-17 PMC5635695 29018125

[48] Kader A, Simm R, Gerstel U, Morr M, Römling U (2006). Hierarchical involvement of various GGDEF domain proteins in rdar morphotype development of *Salmonella enterica* serovar Typhimurium. Mol Microbiol.

[49] Caly DL, Bellini D, Walsh MA, Dow JM, Ryan RP (2015). Targeting cyclic di-GMP signalling: a strategy to control biofilm formation?. Curr Pharm Des.

[50] Reinders A (2015). Expression and genetic activation of cyclic di-GMP-specific phosphodiesterases in. Escherichia coli. J Bacteriol.

[51] Wilde A, Mullineaux CW (2015). Motility in cyanobacteria: polysaccharide tracks and Type IV pilus motors. Mol Microbiol.

[52] Schuergers N, Wilde A (2015). Appendages of the cyanobacterial cell. Life.

[53] Zacharias DA, Violin JD, Newton AC, Tsien RY (2002). Partitioning of lipid-modified monomeric GFPs into membrane microdomains of live cells. Science.

[54] De N (2008). Phosphorylation-independent regulation of the diguanylate cyclase WspR. PLoS Biol.

[55] Stanier RY, Kunisawa R, Mandel M, Cohen-Bazire G (1971). Purification and properties of unicellular blue-green algae (order *Chroococcales*). Bacteriol Rev.

[56] Iwai M, Katoh H, Katayama M, Ikeuchi M (2004). Improved genetic transformation of the thermophilic cyanobacterium, *Thermosynechococcus elongatus* BP-1. Plant Cell Physiol.

[57] Pinto F, Pacheco CC, Ferreira D, Moradas-Ferreira P, Tamagnini P (2012). Selection of suitable reference genes for RT-qPCR analyses in cyanobacteria. PloS one.

