# Supplementary material for: Tlr1612 is the major repressor of cell aggregation in the light-color-dependent c-di-GMP signaling network of Thermosynechococcus vulcanus
Source: Sci Rep. 2018 Mar 28;8:5338. doi: 10.1038/s41598-018-23628-4 (PMC5871770; doi:10.1038/s41598-018-23628-4)
Supplement: Supplementary file 1 — Supplementary Information [file 41598_2018_23628_MOESM1_ESM.pdf]

Supporting Information

Tlr1612 is the major repressor of cell aggregation in the light-color-dependent c-di-GMP signaling network of *Thermosynechococcus vulcanus*

Gen Enomoto[1], Yukiko Okuda[1,2], and Masahiko Ikeuchi[1,2]

[1]Department of Life Sciences (Biology), Graduate School of Arts and Sciences, The University of Tokyo, Komaba 3-8-1, Meguro, Tokyo 153-8902, Japan

[2]Core Research for Evolutional Science and Technology, Japan Science and Technology Agency, 4-1-8 Honcho Kawaguchi, Saitama 332-0012 Japan

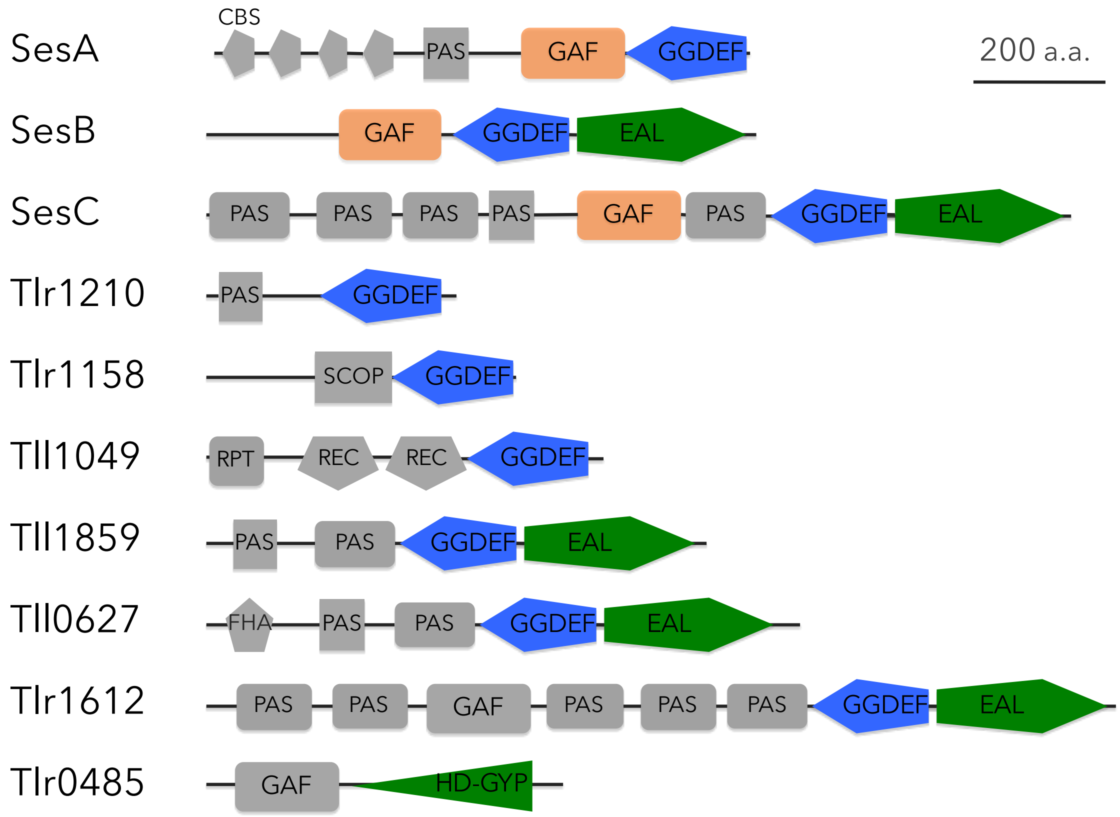

Fig. S1. Domain architecture of the 10 proteins that encode GGDEF/EAL/HD-GYP domains in *Thermosynechococcus*.

Each CBCR-GAF domain is highlighted in orange.

```
SesA          ---LEQANTNLQEMVYIDSLTDIGNRRCFDELFLKEWRRCQ-REQKPLSLIMLDIDCFKA
SesB          ---RKEVEAQLLYQARHDHLTHLPNRWLFEDQVRLSLNHAAAHSDFHYAVLCLDLDRFKT
SesC          ---RKQMEEQLIHDALHDALTGLPNRTLLQERLKHCWRQYQRRRDRPFAVVFIDIDRFKR
Tlr1210       ---LEAENHYLQQISQQDSLTKLANRRTFESRLRLVWQEAA-ATHTPLAIVLLDIDHFKD
Tlr1158       ---MRQREQALLALAWLDPLTGAFNRRFLLSRVEAYESQAA-TGEALLTVMIFDIDHFKR
Tll1049       ---RLERTRLLKRLAEVDPLTGLSNRRKATEDIERFLTLAS-RQQQTLCIAVLDLDYFKR
Tll1859       ---HRLGEAQLLHDGLHDWLTGLANRTLLFDRIEQAARHGRRRPDYKFAILFIDIDRFKV
Tll0627       ---YHLIQEKILHDALHDAMTGLANRVLLIDRLTQAIARRQRRPNSLFAVLFLDVDRFKV
Tlr1612       ---RVQVQMQLHHDAYHDSLTGLPNRLYFMEQLAAAISQAQTNPQFHYAVLFFDLDRFKV
PleD          TDYLRNNLDHSLELAVTDQLTGLHNRRYMTGQLDSLVKRAT-LGGDPVSALLIDIDFFKK
PdeA          ---GVVAPETKFSASELCDLTGLLDRRSFLARARERLAQEG-----THQLVVADLDRLRR
YdeH          ---TDYKIYLLTIRSNMDVLTGLPGRRVLDESFDHQLRNAE-PLN--LYLMLLDIDRFKL
                                 :*   .*                           *:* ::
                                     RXXD motif  GGDEF motif
SesA          YNDHYGHLQGDEILKQVARILESHL-QRAGDLATRFGGEEFALILPDTDQRG-AIHIVEK
SesB          LNDSLGHAIGDLLLQAFAKRLTRCV--SPQDVVARLGSDEFAVLLNDIQGI---EQAQAI
SesC          VNDSLGHQAGDQLLITLAQRMQTVV--RQGDTLARLSGDEFVVLCEDLDPQQMVAQVQAQ
Tlr1210       YNDTYGHLAGDRALQRVAYAIKSQV--RATDLVARYGGEEFVLLLSQAHHDA-AICILER
Tlr1158       INDTYGHDQGDVVLRTLAARAKQVLESCQDGILVRWGGEEFVLLFFVAYEDQ-AKAVAEE
Tll1049       INDTYGHDVGDQVLVTTARMLRQSL--RIEDVVARWGGEEFLIALYGISLEV-AKHRLAI
Tll1859       INDSLGHSSGDLILIELANRLTRIV--RADDTVARIGGDEFVILLDDISDN---NDALAV
Tll0627       INDSLGHLAGDQLLIGIGQRLRACT--RPEDTIARLGGDEFAILLEDLRDP---AQAMKV
Tlr1612       INDSLGHGVGDQVLIAFATWLRRLV--GNQYTVARFGGDEFVILLTHVPHL---QPAIAL
PleD          INDTFGHDIGDEVLREFALRLASNV--RAIDLPCRYGGEEFVVIMPDTALAD-ALRIAER
PdeA          LNEALGHERADLVLAALGSRLAAAF--PAQSILGRIGEDEFAVLCQPLGYEP--------
YdeH          VNDTYGHLIGDVVLRTLATYLASWT--RDYETVYRYGGEEFIIIVKAANDEE-ACRAGVR
               *:  **  .*  *   .                * . :** :

SesA          IQGALAEANITHAKSTVGPKLTASFGIATTIPT-LDHTPEMLLHLADQCLYEAKTHGRDR
SesB          AQRLRERLNHPFEIDHYTLYTTVSIGLVMGDAH--YKSTEELLRDADMAMYYAKSKGHNR
SesC          VTDLERVIQEPLIIDGHLLSVSASIGVAFSDRE--ATSAATLLRDADIAMYQAKKQGLGQ
Tlr1210       IFNHIRSLEIPHASSPVKPYLTLSAGICIATATPRDCPISELIATADAALYEAKRSGRDR
Tlr1158       LCSVVRSQPVELTD-QYFLSVTISLGVTLFRGG--EQVLSDSIPRADQALYQAKHGGRDR
Tll1049       CLQRLRNHTFHAPN-HDSFKVTFSAGISQFPTH--GQSLVQLVKMADDALYAAKAAGRSQ
Tll1859       CDRIHSELDKPFVVNDQPIALRVSIGVALRSAH--IEKAENYLRNADIAMYRAKLAGGNR
Tll0627       AERILMVLGRPFLLEGHEIFTTASIGIAFPWED--SQTAEDLLRDADTAMYRAKSLGKAR
Tlr1612       CEELIHSLQQPLRVGERQIFLSTSIGVVFAQNE--YETGMAVLRDADTAMYAAKRQSGRR
PleD          IRMHVSGSPFTVAHGREMLNVTISIGVSATAGE--GDTPEALLKRADEGVYQAKASGRNA
PdeA          SDVLRSALEQPLRVAGFDIHPTLSIGAVSAEGGLDAPDAAELLRRAELAVEAAAAAGRGG
YdeH          ICQLVDNHAITHSEGHIN--ITVTAGVSRAFPE---EPLDVVIGRADRAMYEGKQTGRNR
                                     : *                :  *:  :  .   .
```

Fig. S2. Amino acid sequence alignment of the GGDEF domains in *Thermosynechococcus vulcanus*.

Conserved amino acid residues in the active site are shaded in red. The conserved Arg and Asp residues in the RXXD motif that are necessary for allosteric product inhibition are shaded in blue. PleD and PdeA of *Caulobacter crescentus* and YdeH/DgcZ of *E. coli* are included for comparison.

```
                                       EAL motif
SesB        DRLHLEVELRQAIDQGDFALYYQPIINLRSQSPRGFEALVRWQK-EDTLISPTVFIPVAE
SesC        SCFTLESQLHQAIANSELQVYFQPIVEMKTGAIVGLEALSRWFDPEKGEISPSEFITLAE
Tll1859     DRLSLEIGLRQAIERDEFTLLYQPIYRLRDNALYGFEALIRWQHPTQGFLLPDRFIPLAE
Tll0627     ALMQMETELRRAAEREEFLVYYQPIVELATLKMVGFETLLRWQHPERGIISPGEFMAVAE
Tlr1612     ERLHLEHDLRQALNQGGLQLLYQPVVDLQSQQLVGMEALVRWQHPERGLLSPAHFIPIAE
PdeA        SRLALEADLRGAIGRGEITPYFQPIVRLSTGALSGFEALARWIHPRRGMLPPDEFLPLIE
YahA        HHIVTPEAISLALENHEFKPWIQPVFCAQTGVLTGCEVLVRWEHPQTGIIPPDQFIPLAE
              :     :  *  .  :    **:         * *.* ** .     : *  *:.: *

SesB        ETGLIFELSRWVLRSACEQLQQWQKRYPKLRSLGFTVSINLSANQFSLPTLVAEIEQALE
SesC        QAGLIVSLGRQVLERAIQEFSQWRQQDSRRQTMT--LGINISPQQLVDANFVSDILAALR
Tll1859     ETGLILPIGDWVIWRACRDLQYWHEQFPQC-QLS--VNVNLSNRQLMHPALPEQVLAALE
Tll0627     ETGLILPISWWVMAEACRQMQRWAVIFPHSRKLR--IGVNLSGRHFQQADLLPNLRSILA
Tlr1612     DTGLIVALDQWALAQACWQLWTWRQQYSTAADL--VLSVNVSAKTLQDPTFLQHLDTIRQ
PdeA        EMGLMSELGAHMMHAAAQQLSTWRAAHPAMGNLT--VSVNLSTGEIDRPGLVADVAETLR
YahA        SSGLIVIMTRQLMKQTADILMPVKHLLPDNFHIG----INVSAGCFLAAGFEKECLNLVN
            . **:  :    :  :   :       .    :     :*:*   :  . :  .

SesB        HHHLAGQFLKIEITESTLMHHLDSACEILTKLKAMGVRINIDDFGTGYSSLSYLRNLPLD
SesC        RAQLPPHLLHLEITETTMIRNLEATLQVAEKLQQLGVALNIDDFGTGYSSLSRLHQLPIH
Tll1859     QTQIPPHCLHLEMTESVGIDQPDQVRETLCKLKAQGIKLSLDDFGTGYSSLRYLTHLPID
Tll0627     ETGFPAQRLCLEVTEGILIDNKEVAIATLEEIRAMGIGVSMDDFGTGYSSLSYLHRFPID
Tlr1612     RYPLPKGQLLLELTERIGIELGSEMLSLLESLRHRHVEISIDDFGTGYSCLSYLHSLPIQ
PdeA        VNRLPRGALKLEVTESDIMRDPERAAVILKTLRDAGAGLALDDFGTGFSSLSYLTRLPFD
YahA        KLGNDKIKLVLELTERNPIPVTPEARAIFDSLHQHNITFALDDFGTGYATYRYLQAFPVD
                    * :*:**   :            ::     . :******::    *  :*..

SesB        GIKIDRSFISQMDRSQEDLELVRTILVLARNLHLDCIAEGIESTTQLQLLRSLRCPFGQG
SesC        ALKIDRSFVQSLEESQAAQEIIGAVIALGKSLRLDVVAEGVETAVQATQLMDLGCLYGQG
Tll1859     ILKVDRSFVKLITETEQRPLVIDAIVSLAKGLALEVVAEGVEHPYQVTRLRELGCDYVQG
Tll0627     TLKIDRTFISALNSEESSATIVHAILMLAHSLRLQVVAEGIETREQYRALQALGCNYGQG
Tlr1612     HLKVDRSFVSQLEENERNLQIIRMILLLSKQLGYRVIAEGIETPKQLQILQELGCDEGQG
PdeA        TLKIDRYFVRTMGNNAGSAKIVRSVVKLGQDLDLEVVAEGVENAEMAHALQSLGCDYGQG
YahA        FIKIDKSFVQMASVDEISGHIVDNIVELARKPGLSIVAEGVETQEQADLMIGKGVHFLQG
             :*:*: *:           ::  :: *.:      :***:*       :        **

SesB        YLFAPPLTADK
SesC        YFFYPPLPIDR
Tll1859     YYFSRPLTTEQ
Tll0627     YFFARPLSARQ
Tlr1612     YLFARPLPPET
PdeA        FGYAPALSPQE
YahA        YLYSPPVPGNK
            : :  .:.
```

Fig. S3. Amino acid sequence alignment of the EAL domains in *Thermosynechococcus vulcanus*.

The conserved amino acid residues in the active site are shaded in red. PdeA of *Caulobacter crescentus* and YahA/PdeL of *E. coli* are included for comparison.

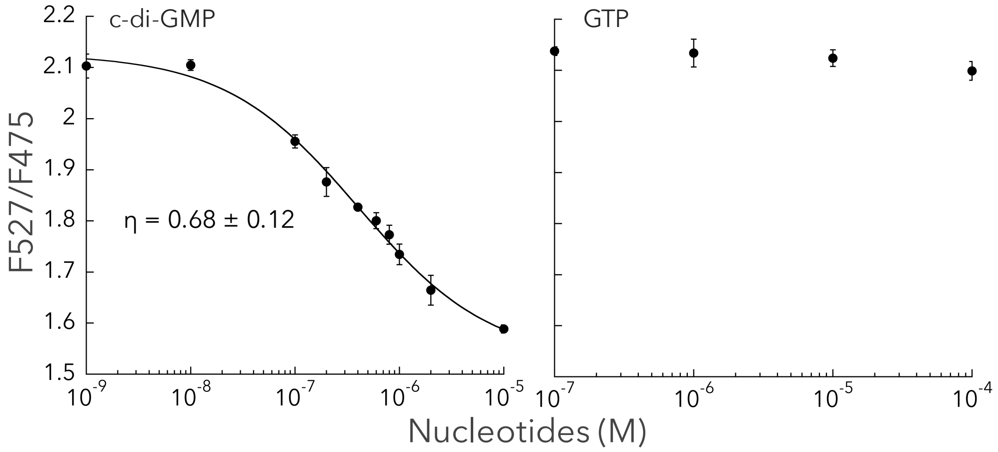

Fig. S4. FRET-based biosensor assay for assessing c-di-GMP binding by *E. coli* YcgR.

The nucleotide concentration–response curve for the FRET efficiency (YFP emission/ CFP emission; F527/F475) for c-di-GMP (left) and GTP (right). Data represent the mean ± SD of three independent experiments. The Hill coefficient (η) was estimated by curve fitting.

Table S1 Oligonucleotides used for plasmid construction.

Purpose	Name	Sequence (5'→ 3')
CyPet (A206K)	CyPet-1F	CAATCTAAATTATCTAAAGATCCAAACGAAAAG
	CyPet-2R	AGATAATTTAGATTGAGTGGATAAGTAATGGTT
YPet (A206K)	YPet-1F	CAATCTAAATTATTCAAAGATCCAAACGAAAAG
	YPet-2R	GAATAATTTAGATTGATAGGATAAGTAATGGTT
FRET-biosensor	pET28TEV-1R	CATATGGCTCTGAAAATACAG
	PET28_R1	GGATCCGAATTCGAGCTC
	CyPet-3FTEV	TTTCAGAGCCATATGTCTAAAGGTGAAGAA
	CyPet-4R	TTTGTACAATTCATCCATAC
	YPet-3F	TCTAAAGGTGAAGAATTA
	YPet-4R28a	CTCGAATTCGGATCCTTATTTGTACAATTCATTCATAC
	ycgR-1FCYP	GATGAATTGTACAAAACTAGTATGAGTCATTACCATGAGC
	ycgR-2RYPe	TTCTTCACCTTTAGAGGTACCGTCGCGCACTTTGTCCGC
	Tll0007-15FCYP	TTGTACAAAACTAGTCCGAGTCGGCGCCAAAG
	Tll0007-16RYPe	ACCTTTAGAGGTACCACCATAAAGCAGTTGTACC
Tll0007 (R605A)	tll0007-32F	AAGCGATGCCTTTCCCGTTTGCACTGTG
	tll0007-33R	GGAAAGGCATCGCTTTGGCGCCGACT
SesA (R676A)	tlr0924-28F	ATTTGCAGGCGGCAGGGGATTTGGCCAC
	tlr0924-29R	CTGCCGCCTGCAAATGGCTTTCCAAAA
Δ*tlr1210*	tlr1210-14F1R	AACGACGGCCAGTGACTCAATTCCATCGAGGCGAT
	tlr1210-15Rup	GCGAGCAGGGGAATTAGACTGACTCAGGACTCAAG
	tlr1210-16Fcom	CCGCTTCGCAATATTAGAAGTCCTTCATTGCAGTGG
	tlr1210-7Rrrn	CAGACCGCTTCTGCGCTACGGGGCCTTTTCCGAAG
Δ*tlr1158*	tlr1158-1F1R	AACGACGGCCAGTGAAGATCAGAGGCAGGGTCAC
	tlr1158-2Rup	GCGAGCAGGGGAATTGCCCTAACCAGAATCGATTG
	tlr1158-3Fcom	CCGCTTCGCAATATTGCTAAAAAAAGTGCAGGAAC
	tlr1158-4R2F	GAAACAGCTATGACCGCGGCGACTCAATCATATC
Δ*tll1049*	tll1049-1F1R	AACGACGGCCAGTGATGGGAGAACTTTGGGTACAG
	tll1049-2Rup	GCGAGCAGGGGAATTACCTAGCCGCCAATAGCCC
	tll1049-3Fcom	CCGCTTCGCAATATTTGGCCATTTTGCTTTATCAC
	tll1049-4R2F	GAAACAGCTATGACCGCTTATTGCTCTTGGGAGGA
Δ*tll1859*	tll1859-1F1R	AACGACGGCCAGTGAGCTCATAGGTGGTGAAACGA
	tll1859-2Rup	GCGAGCAGGGGAATTAAAATATCCTAGCTTAAGTGC
	tll1859-3Fcom	CCGCTTCGCAATATTGGAGCAGGATGACTCACCT
	tll1859-4R2F	GAAACAGCTATGACCTTGTCTTTCTAGGGGGTGTG
Δ*tll0627*	tll0627-1F1R	AACGACGGCCAGTGATTCAGAATGCTGTCCTTGGC
	tll0627-2Rup	GCGAGCAGGGGAATTATCAATGGCCTCGGCAAGGA
	tll0627-3Fcom	CCGCTTCGCAATATTGAAGTCTGATCTGCCGAAGA
	tll0627-4R2F	GAAACAGCTATGACCGTTTCCGCCAAAATAGAC
Δ*tlr1612*	tlr1612-1F1R	AACGACGGCCAGTGATCTTTGGCTCCCAGGCGAT
	tlr1612-2Rup	GCGAGCAGGGGAATTGGAAAGCCGCTTGTAAAGCT
	tlr1612-3Fcom	CCGCTTCGCAATATTAGGATGGTTCCTCACCACGCT
	tlr1612-4R2F	GAAACAGCTATGACCTCCCAATCCTCAAGATTG
Δ*tlr0485*	tlr0485-1FpS	CCGGGGGATCCGCCCGTGGCGATTGACTCTTTG
	tlr0485-2RCm16	CCGGACATCAGCGCTTCCTCTAGGGATTGACTG
	tlr0485-3FCm15	ACGGTTAGCAGGCCTATCCTGATCTGGTGGAAC
	tlr0485-4RpS	CGGCCGCTCTAGCCCCTATGGGATGGCAATGG

Table S2 Oligonucleotides used for Real-time qPCR analysis.

Target	Name	Sequence (5'→ 3')
rnpB	TrnpB-3F	TGGAGGGAAACCTCACAGGC
	TrnpB-4R	CGCTAGCACACCTTGCTTCC
sesA	tlr0924-32F	GGGCCAAAACTAACGGCCAG
	tlr0924-33R	GATCCGCGAGATGCAACAGC
sesB	tlr1999-28F	TGGCTCGCAATCTCCACCTC
	tlr1999-29R	AGGTAGCCTTGCCCAAAGGG
sesC	tlr0911-23F	GGTGCAAGCGCAGGTTACTG
	tlr0911-24R	GCCAATACTGGCACTCACGC
tlr1210	tlr1210-17F	GGCGCGATCGCTATGTGTTG
	tlr1210-18R	TACGGGGCCTTTTCCGAAGG
tlr1158	tlr1158-9F	GGCATTTTGGTACGCTGGGG
	tlr1158-10R	AGGCTGGCTTCTCACCACTG
tll1049	tll1049-9F	CTAGCCGCCAACAGCAAACC
	tll1049-10R	GATCGCCAACATCGTGACCG
tll1859	tll1859-9F	GCTCATCACCGAAACCGAGC
	tll1859-10R	TGCTCCACCCCTTCAGCAAC
tll0627	tll0627-11F	CCGAACGGGGCATCATTTCC
	tll0627-12R	AGCCTCTGCCATAACCCACC
tlr1612	tlr1612-9F	TTGGCAGCTTTGGACTTGGC
	tlr1612-10R	TCGTGTCAAGGTGCTGGAGG
tlr0485	tlr0485-10F	TTCCTTGGACACCCCACCTG
	tlr0485-11R	ATTTCGGCGGCACCAATACC
